# *Bradyrhizobium diazoefficiens* USDA110 PhaR functions for pleiotropic regulation of cellular processes besides PHB accumulation

**DOI:** 10.1186/s12866-018-1317-2

**Published:** 2018-10-24

**Authors:** Shogo Nishihata, Takahiko Kondo, Kosei Tanaka, Shu Ishikawa, Shinji Takenaka, Choong-Min Kang, Ken-ichi Yoshida

**Affiliations:** 1 Department of Agrobioscience, Kobe University, 1-1 Rokkodai, Nada, Kobe, 657 8501 Japan; 2 Department of Science, Technology and Innovation, Kobe University, 1-1 Rokkodai, Nada, Kobe, 657 8501 Japan; 3 Department of Biological Science, California State University, Stanislaus, Turlock, CA 95382 USA

**Keywords:** *Bradyrhizobium diazoefficiens*, Poly-3-hydroxybutyrate (PHB), Transcriptome, Transcription factor, DNA binding

## Abstract

**Background:**

*Bradyrhizobium diazoefficiens* USDA110 nodulates soybeans for nitrogen fixation. It accumulates poly-3-hydroxybutyrate (PHB), which is of physiological importance as a carbon/energy source for survival during starvation, infection, and nitrogen fixation conditions. PHB accumulation is orchestrated by not only the enzymes for PHB synthesis but also PHB-binding phasin proteins (PhaPs) stabilizing the PHB granules. The transcription factor PhaR controls the *phaP* genes.

**Results:**

Inactivation of *phaR* led to decreases in PHB accumulation, less cell yield, increases in exopolysaccharide (EPS) production, some improvement in heat stress tolerance, and slightly better growth under microaerobic conditions. Changes in the transcriptome upon *phaR* inactivation were analyzed. PhaR appeared to be involved in the repression of various target genes, including some PHB-degrading enzymes and others involved in EPS production. Furthermore, in vitro gel shift analysis demonstrated that PhaR bound to the promoter regions of representative targets. For the *phaP1* and *phaP4* promoter regions, PhaR-binding sites were determined by DNase I footprinting, allowing us to deduce a consensus sequence for PhaR-binding as TGCRNYGCASMA (R: A or G, Y: C or T, S: C or G, M: A or C). We searched for additional genes associated with a PhaR-binding sequence and found that some genes involved in central carbon metabolism, such as *pdhA* for pyruvate dehydrogenase and *pckA* for phosphoenolpyruvate carboxykinase, may be regulated positively and directly by PhaR.

**Conclusions:**

These results suggest that PhaR could regulate various genes not only negatively but also positively to coordinate metabolism holistically in response to PHB accumulation.

**Electronic supplementary material:**

The online version of this article (10.1186/s12866-018-1317-2) contains supplementary material, which is available to authorized users.

## Background

Poly-3-hydroxybutyrate (PHB) is a type of polyhydroxyalkanoate (PHA), which accumulates within the cells of various microorganisms as an energy storage substance under stress conditions, such as oxygen limitation and nutrient starvation [1]. PHB, a potential substitute for petroleum-based plastics, is produced in an enzymatic process involving reactions requiring mild conditions and is biodegradable even under opportunistic anaerobic conditions [2].

PHB or PHA accumulation is of physiological importance in many bacterial species. *Azospirillum brasilense* is a free-living soil bacterium that affects the growth of numerous agricultural crops [3]. Mutant strains of *A. brasilense* lacking the ability to accumulate PHA exhibited decreased resistance to various stress conditions, and degradation products of PHB could protect microorganisms from hydroxyl radicals [4, 5]. In the case of free-living *Sinorhizobium meliloti*, an alfalfa symbiont, PHB is continually synthesized and degraded, which may serve as an energy source under starvation conditions [6]. In addition, a mutant defective in PHB synthesis demonstrated a decreased symbiotic ability and diminished nitrogen fixing ability with weakened nitrogenase activity [7]. In *Bradyrhizobium japonicum* (*Bradyrhizobium diazoefficiens*) USDA110, a soybean symbiont bacterium, under the symbiotic state, it was proposed that utilization of PHB might maintain nitrogen fixation activity [8]. In contrast, a mutant of *B. diazoefficiens* USDA110 defective in PHB accumulation had a better symbiotic performance than the wild type [9]. These observations suggest that the physiological importance of PHB accumulation varies from species to species [10]. Though it has been proposed that PHB is used as an energy source during the bacterial differentiation process from the free-living form to the symbiotic form [11], there remain many unclear points about the physiological significance of PHB.

PHB metabolism in *S. meliloti* has been elucidated [10]; synthesis of PHB proceeds thusly: first, PhbA (Ketothiolase) synthesizes acetoacetyl-CoA from two molecules of Acetyl-CoA. Next, PhbB (Acetoacetyl-CoA reductase) reduces Acetoacetyl-CoA to form 3-hydroxybutyryl-CoA. Then, PhbC (PHB synthase) polymerizes 3-hydroxybutyryl-CoA to form PHB. Degradation of PHB proceeds as such: PHB is monomerized by PhaZ (PHB depolymerase) into 3-hydroxybutyrate, which is further oxidized by BdhA (3-hydroxybutyrate dehydrogenase) to produce acetoacetate. Esterification of acetoacetate by AcsA2 (acetoacetyl-CoA synthetase) produces acetoacetyl-CoA. Finally, acetoacetyl-CoA is decomposed to acetyl-CoA. In *B. diazoefficiens* strain USDA110, which also accumulates PHB in large quantities, PHB metabolism was deduced by genomic comparison with *S. meliloti*, and transcription of the paralogous genes involved in PHB metabolism was analyzed [12]. The genes transcriptionally induced during PHB accumulation in the free-living state were *phbA2* (*bll0226*), *phbB2* (*bl10225*), *phbC3* (*bll4360*), *phbC5* (*bll6073*), and *phaZ1* (*blr0908*) [12]. In addition, *phbC3* notably contributed to PHB synthesis, since its inactivation abolished PHB accumulation [9].

*Ralstonia eutropha* was studied intensively to elucidate the mechanisms controlling PHB granule stabilization [13], which requires PhaR and phasin proteins (PhaPs) [14]. When the PHB level is low, the PhaR repressor binds to the promoter regions of its own gene and *phaP* genes to minimize their expression. PhaR binds not only to DNA but also to PHB. Once PHB production is commenced, the intracellular concentration of PHB reaches a certain level that antagonizes DNA binding of PhaR, and PhaR dissociates from the promoter regions. At the initial stage of PHB accumulation, PhaR binds to PHB granules instead of DNA, and transcription of the *phaP* promoters is elevated as PhaR dissociates from DNA to induce PhaP synthesis. Since PhaPs have a higher affinity for PHB than PhaR, the increasing amount of PhaPs induces dissociation of PhaR from PHB. PhaPs are amphipathic and stabilize hydrophobic PHB granules. Finally, PhaR released from PHB binds once again to the promoter regions to repress transcription of the *phaR* and *phaP* genes. Repeating this process, PHB accumulates in the cytoplasm as large stabilized granules. In *B. diazoefficiens* USDA110, PHB accumulation is achieved in a similar way as in *R. eutropha* [15]. There are at least four PhaP paralogs and PhaR, which bound to PHB in vitro; PhaP4 exhibited the highest affinity to PHB, which could compete with PhaR to induce its dissociation from PHB [12].

Disruption of *phaR* in *R. eutropha* led to a drastic reduction in PHB accumulation [13, 14, 16]. In addition, *phaR* inactivation in *Methylobacterium extorquens* resulted in slower proliferation and a decrease in PHB accumulation [17]. Furthermore, under anaerobic conditions, disruption of *aniA* (*phaR*) in *S. meliloti* reduced PHB accumulation but increased the accumulation of exopolysaccharides (EPS) [18]. In addition, *aniA* (*phaR*)-disruption in *Rhizobium etli* [19] and *B. diazoefficiens* [9] produced similar phenotypes as the *S. meliloti* mutant, even under aerobic conditions. In the present study, we constructed and investigated a *phaR*-inactive mutant of *B. diazoefficiens* USDA110. Our transcriptomic analysis reveals that PhaR plays an important role not only in PHB accumulation but also in pleiotropic regulation. Furthermore, we analyzed DNA binding of a recombinant PhaR with a C-terminal His_6_-tag fusion in vitro, and deduced a consensus sequence for PhaR binding, providing a holistic view of PhaR regulon.

## Methods

### Bacterial strains, plasmids, culture conditions, and oligonucleotide primers

Bacterial strains and plasmids used in this study are listed in Table 1. Strains of *Escherichia coli* were cultured in lysogeny broth (LB) [20] at 37 °C with shaking at 180 rpm. Strains of *B. diazoefficiens* were maintained on peptone salts yeast extract medium (PSY) [21] at 28 °C. A fresh colony was selected from a media plate and was inoculated into test tubes containing 5 mL of PSY and precultured at 28 °C with shaking. An aliquot of the culture was diluted in 5 mL of yeast extract mannitol medium (YEM) [22] or tryptone yeast medium (TY) [23] and cultured further at 28 °C with shaking. When needed, polymyxin B (Pm) and kanamycin (Km) were added at 50 μg/mL, as noted in the following procedures. For cultivation under microaerobic conditions, plates were sealed inside an AnaeroPouch-Anaero anaerobic gas generator system (Mitsubishi Gas Chemical) and incubated at 28 °C. The oligonucleotide primers used in this study are listed in Additional file 1: Table S1.

**Table 1:** Bacterial strains and plasmids used in this study

Strains or plasmids	Derivation and relevant properties	Source of reference
*B. diazoefficiens*		
USDA110	Wild type	[42]
ΔphaR	A mutant of USDA110 with deletion of *phaR*	This work
*E. coli*		
DH5α	*supE44* Δ*lacU169 hsdR17 recA1 endA1 gyrA96 thi-1 relA1*	Takara Bio
S17–1	F- *thi pro hsdR* [RP4–2 *tet*::Mu *kan*::Tn7 (*trp str*)]	[43]
BL21(DE3)	F^−^ *ompT hadS*_β_ (rβ^−^ mβ^-)^ *gal dcm* (DE3)	Takara Bio
Plasmids		
pK18m*obsacB*	Mobilizable plasmid containing *oriV*, *oriT*, *mob*, *sacB*, and *kan*	[44]
pK18m*obsacB*Δ*phaR*	pK18 m*obsacB* containing the flanking regions of *phaR*	This work
pET28b	*kan*	Takara Bio
pET28PhaR	pET28b carrying the coding region of *phaR*	This work
pMD20	A linearized vector for TA cloning of PCR fragments	Takara Bio

### Mutant construction

Two DNA fragments were amplified from the chromosomal DNA of *B. diazoefficiens* USDA110 by PCR using the primer pairs FFphaR/FRphaR and RFphaR/RRphaR (Additional file 1: Table S1). The fragments were fused by a recombinant PCR reaction using the primer pairs FFphaR/RRphaR (Additional file 1: Table S1). The fusion PCR product, that corresponds to a mutated *phaR* with an internal deletion from the 21st to 185th codons, was trimmed by two restriction enzymes, XbaI and HindIII, ligated into plasmid pK18mobsacB (Table 1) cleaved previously with the same enzymes, and transformed into *E. coli* DH5α. Correct construction of the recombinant plasmid was confirmed by DNA sequencing. The plasmid was transferred into *E. coli* S17–1, which was then cultured overnight at 37 °C as the plasmid donor. USDA110 as the recipient was cultured with Pm at 28 °C until the OD_600_ = 1.0. Equal volumes of the donor and the recipient cultures were mixed and spun down. The collected cells were suspended in sterilized water and pipetted onto a membrane filter (Merck Millipore) placed on a PSY agar plate without antibiotics and incubated overnight at 30 °C. Following this incubation, the bacterial cells on the filter were suspended in sterilized water, spread on PSY agar plates containing both Pm and Km, and incubated at 28 °C to form colonies. Chromosomal DNA of some colonies was subjected to PCR using the primers FFphaR and RRphaR to choose a candidate clone having the entire plasmid DNA integrated into the target site of the chromosome. The chosen clone was cultured in PSY containing Pm alone to induce a pop-out event—spontaneous intra-chromosomal recombination of the plasmid DNA encoding Km resistance and *sacB* genes—and plated on PSY containing both 10% sucrose and Pm. The resulting colonies were duplicated on two plates, one containing Km and the other Pm. A clone losing Km resistance, whose correct construction was confirmed by PCR analysis and DNA sequencing with primers phaR-c-F and phaR-c-R (Additional file 1: Table S1), was designated as strain ΔphaR with deletion of *phaR*.

### Phenotypic analysis

To determine PHB accumulation, bacterial cells (1 OD_600_ unit) were collected and suspended in 5% sodium hypochlorite and incubated overnight at 37 °C. After a brief centrifugation, the precipitate was suspended in distilled water, and mixed with a twofold volume of 99.5% ethanol and acetone 1:1 mixture. After another spinning down, the precipitant was dissolved in chloroform at 50 °C. The solution was transferred to another tube and air-dried. After addition of 95% sulfuric acid, the tube was incubated at 100 °C for 20 min to lyse the cells, and OD_235_ of the solution was measured to calculate the amount of PHB. A standard curve was prepared by using various amounts of PHB (Sigma-Aldrich) as previously described [24].

To evaluate EPS production, bacterial cells (1 OD_600_ unit) were spun down, and the supernatant mixed with a threefold volume of ethanol, chilled at − 20 °C for 10 min, and centrifuged again. The precipitated EPS was air-dried, suspended in water, mixed with 0.2% anthrone (Nacalai Tesque) in 95% sulfuric acid, incubated at 100 °C for 10 min, and cooled under running water. The OD_620_ of the solution was measured to calculate the amount of EPS as previously described [25].

To measure the intracellular glycogen, bacterial cells were collected and suspended in 30% KOH and heated at 100 °C for 20 min. After adding threefold volume of ethanol, the tube was centrifuged and the supernatant discarded. The precipitate was suspended in water, mixed with an equal volume of 0.2% anthrone in 95% sulfuric acid, incubated at 100 °C for 10 min, and cooled under running water. The OD_620_ of the solution was measured, and a calibration curve was prepared using glucose to calculate the amount of glycogen as described [26].

Stress tolerance was tested as follows: bacterial strains were precultured in TY and YEM to stationary phase. For the heat shock stress experiments, diluted cultures were incubated at 50 °C for 10 min with shaking. For the osmotic stress experiments, cells were mixed with glucose, NaCl, or sucrose at various concentrations and then incubated at 28 °C for 24 h with shaking. Following incubation, the cultures were serially diluted, and aliquots were spotted on PSY agar plates and incubated at 28 °C.

### RNA preparation and analysis

Strains of *B. diazoefficiens* were grown in 50 mL of TY at 28 °C with shaking until the OD_600_ = 0.4. Cells from these cultures were harvested and suspended in 1 mL of LETS buffer (100 mM LiCl, 10 mM EDTA, 10 mM Tris–HCl at pH 7.4), vortex mixed with 0.5 mL of glass beads (φ0.5 mm) and 1 mL of phenol for 6 min, and centrifuged. After centrifugation, 1 mL of the upper aqueous layer was mixed with 1 mL of phenol-chloroform for 30 s. After another centrifugation, 0.75 mL of the upper aqueous layer was mixed with 0.1 mL of 1 M LiCl and 2.5 mL of ethanol and centrifuged. The supernatant was discarded, and the precipitate was dissolved in 0.175 mL of distilled water, mixed with 3.5-fold volume of 4 M sodium acetate (pH 6.0), incubated at − 20 °C for a minimum of 1 h, and centrifuged. The RNA precipitate was dissolved in distilled water, and treated with DNase I at 37 °C for 1 h. Then DNA-free RNA was purified using RNeasy Mini Kit (Qiagen). Concentrations of the purified RNA samples were determined with Nano Vue Plus (GE Healthcare), and the samples were stored in aliquots at − 80 °C prior to the usage.

The mRNA transcription start points were determined by the 5′-RACE method using the 5′-Full RACE Core Set (Takara Bio). 50 ng of RNA was used as the template for reverse transcription, producing the first cDNA strand from the specific primers, RT-primer phaP1-phos and RT-primer phaP4-phos (Additional file 1: Table S1). After treatment with RNase H, the resulting single stranded cDNA was incubated with T4 RNA ligase overnight at 15 °C. The ligated cDNA served as the template in the subsequent nested PCRs with the following primer pairs: phaP1S1/phaP1A1 followed by phaP1S2/phaP1A2 for *phaP1*; and phaP4S1/phaP4A1 followed by phaP4S2/phaP4A2 for *phaP4* (Additional file 1: Table S1). The resulting PCR fragment was cloned into a pMD20 (Takara Bio) and sequenced to identify the transcription initiation point.

For quantitative RT-PCR, 10 ng of RNA was used as a template for reverse transcription using a ReverTraAce qPCR RT Kit (Toyobo) and each of the reverse primers (Additional file 1: Table S1; RT primer pairs were systematically designated beginning with “RT-,” followed by the respective gene name, and “-F” or “-R” at the end). Reverse transcription products were subjected to relative quantification PCR using Thunderbird Sybr qPCR Mix (Toyobo) and the specific primer pairs (Additional file 1: Table S1) in a Thermal Cycler Dice Real Time System MRQ (Takara Bio).

For RNA-sequencing transcriptome analysis, the total RNA was treated using a Ribo-Zero rRNA Removal Kit (Bacteria) (Illumina) to eliminate rRNA, treated with an Agilent RNA Pico kit (Agilent Technologies), and analyzed for purity using a 2100 Bioanalyzer (Agilent Technologies). The mRNA was converted into a cDNA library by using a NEBNext Ultra Directional RNA Library Prep Kit for Illumina (New England Biolabs), NEB Next Multiplex Oligos for Illumina Index Primers Set I (New England Biolabs), and an Agencourt AMPure XP PCR purification system (Beckman Coulter). Quality of the cDNA library was evaluated using an Agilent High Sensitivity DNA Kit and 2100 Bioanalyzer (Agilent Technologies). The cDNA library was subjected to sequencing using a MiSeq Reagent kit v 3 (150 cycles) (Illumina) in Illumina MiSeq (Illumina).

### Databases and software

RNA sequencing data were analyzed using CLC Genomics Workbench version 6.5.1 (Qiagen) as follows. The complete genome sequence of *B. japonicum* (*diazoefficiens*) USDA110 was obtained as a reference from the NCBI database (accession number NC_004463.1). Quality-filtered sequencing reads were aligned to the reference genome sequence. Mapping was based on a minimal length of 100 bp with an allowance of up to two mismatches. Relative transcript abundance was measured in reads per kilobase per megabase of library size (RPKM). Results of the analysis were submitted to the DDBJ Sequence Read Archive (DRA) database under accession number DRA005621. Gene functions were predicted using the Kyoto Encyclopedia of Genes and Genomes (http://www.genome.jp/kegg/) and rhizobase (http://genome.microbedb.jp/rhizobase/). PhaR-binding sequences were predicted by GLAM2 version 1056 (http://meme-suite.org/tools/glam2) in MEME suite version 4.11.2 (http://meme-suite.org/) to detect a motif for consensus sequence under the default settings.

### Preparation of PhaR-His_6_

*B. diazoefficiens phaR* was expressed in *E. coli* BL21(DE3) to prepare PhaR with a C-terminal His_6_-tag (PhaR-His_6_) as follows: a PCR fragment of the *phaR*-coding region was amplified from the DNA of USDA110 using the primer pair pET28b-phaR-gib-F/pET28b-phaR-gib-His6-R (Additional file 1: Table S1). Another PCR fragment was made from plasmid pET28b (Table 1, Takara Bio) with the primer pair pET28b-inverse NcoI-F/pET28b-inverse BamHI-R (Additional file 1: Table S1). This fragment was ligated with the *phaR* fragment using a Gibbson assembly kit (New England Biolabs) to obtain the plasmid pET28PhaR (Table 1), the correct construction of which was confirmed by sequencing. pET28PhaR was introduced into BL21(DE3), which was cultured in LB containing Km until the OD_600_ reached 0.6, and *phaR* was induced by the addition of 1 mM IPTG for 4 h. The cells were harvested and suspended in a buffer (50 mM phosphate at pH 8.0, 20% glycerol, and 0.5 M NaCl), and disrupted by sonication in an ice bath. From the lysate, PhaR-His_6_ was purified using TALON Metal Affinity Resin (Takara Bio).

### Gel shift assay and DNase I footprinting

DNA fragments used for the gel shift assay were PCR fragments amplified from the DNA of USDA110 using the specific primer pairs (Additional file 1: Table S1; They were systematically designated beginning with “EMSA-”, followed by the respective gene name, and “-F” or “-R” at the end). The DNA fragments were combined in the binding-reaction mixture containing 285 mM Tris–HCl (pH 8.0), 0.14 mg/mL bovine serum albumin, 1.43 mM dithiothreitol, 5% glycerol, 0.04 mg/mL poly-deoxyinosinic-deoxycytidylic acid (Merck), and 3.06 mM EDTA. Addition of serially diluted PhaR-His_6_ yielded a total reaction volume of 10 μL, which was incubated for 30 min at 25 °C. The reaction was then mixed with 80% glycerol and applied to 6% PAGE in TAE buffer (pH 8.2) at 100 V and 20 mA for 2 h. The gel was stained with SYBR Green, and the DNA was visualized using the Gel Doc XR+ system (Bio-Rad).

For DNase I footprinting analysis, DNA fragments were differentially labeled at the 5′-teminus on either strand of DNA using PCR with FAM-labeled primers (Additional file 1: Table S1, the primers designated beginning with FAM were labeled at 5′-end). For labeling the upper and lower strands of the *phaP1* promoter region fragment, the primer pairs FAM-phaP1-F/EMSA-phaP1-R and EMSA-phaP1-F/FAM-phaP1-R were used, respectively. For labeling the upper and lower strands of the *phaP4* promoter region fragment, the pairs of FAM-phaP4-F/EMSA-phaP4-R and EMSA-phaP4-F/FAM-phaP4-R were used, respectively. Various amounts of purified PhaR-His_6_ and each DNA fragment were combined in the binding reaction and incubated for 30 min at 25 °C. Subsequently, the mixture was treated with 0.025–0.1 U/μl DNase I (Takara Bio) for 5 min. The reaction was terminated by the addition of EDTA, and the DNA in the mixture was purified using a QIAquick Nucleotide Removal Kit (Qiagen). Corresponding sequence ladders were prepared using a Thermo Sequenase Dye Primer Manual Cycle Sequencing Kit (Thermo Fisher Scientific) according to the standard procedure. The fragment analysis was outsourced to Sigma-Aldrich, Japan.

## Results

### Phenotypic changes caused by inactivation of phaR

**Fig. 1:**
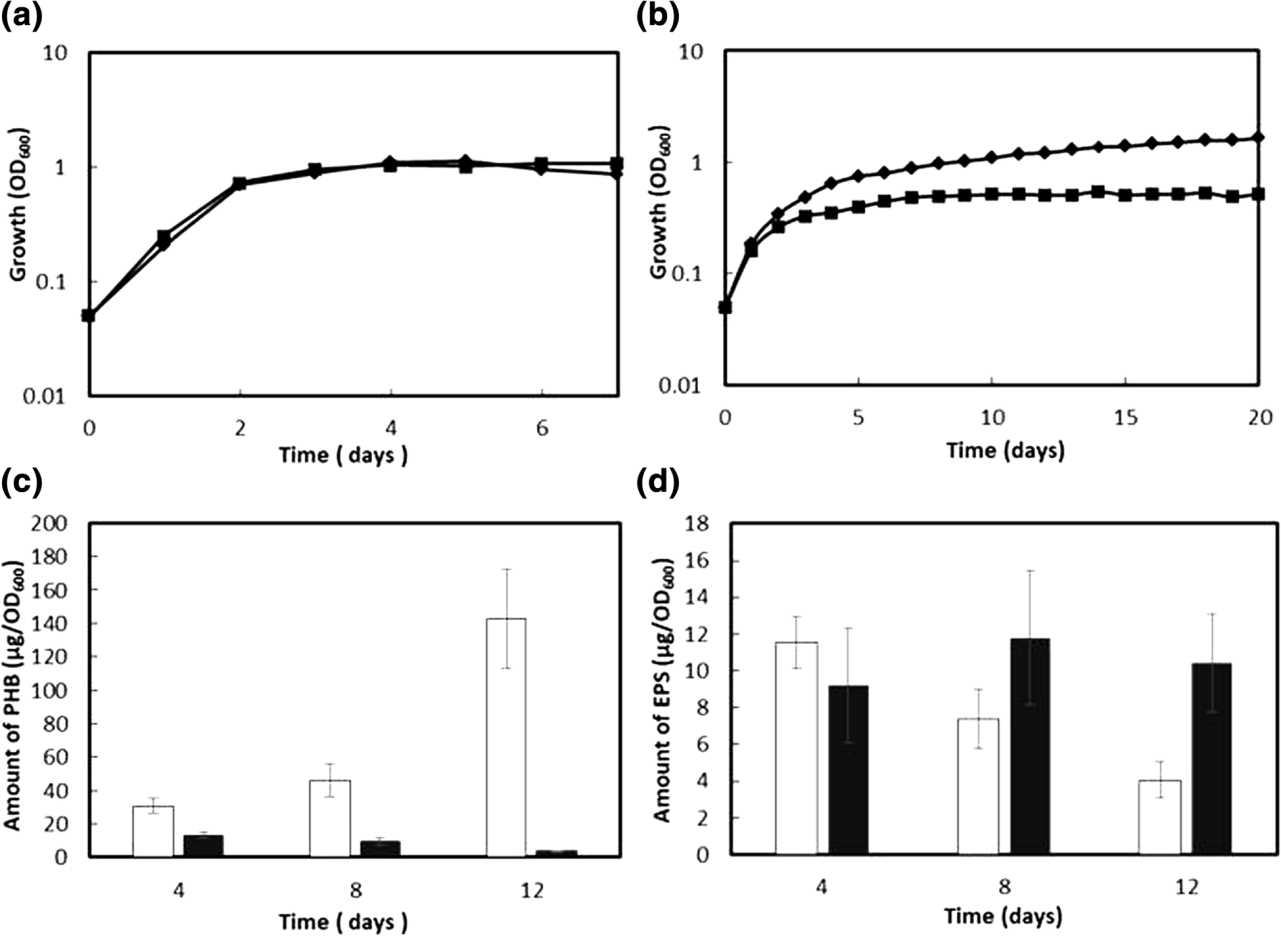
Physiological characterization of ΔphaR. **a** and **b** Growth curves of strains USDA110 (solid diamonds) and ΔphaR (solid squares) of *B. diazoefficiens* grown in TY (**a**) and YEM (**b**). A set of representative results from three independent experiments is shown. **c** PHB accumulation in USDA110 (open bar) and ΔphaR (solid bar) grown in YEM. Values are means ± standard deviation of three independent experiments. **d** EPS production in USDA110 (open bar) and ΔphaR (solid bar) grown in YEM

Two strains of *B. diazoefficiens*, the wild-type USDA110 and its mutant ΔphaR, were grown in TY and YEM (Fig. 1a and b, respectively). TY is rich in nitrogen sources, and USDA110 grown in TY does not accumulate PHB [12]. When grown in TY, there were no obvious differences in the growth of the two strains in 7 days (Fig. 1a). In YEM, which has a higher carbon/nitrogen ratio, USDA110 is known to accumulate PHB [12]. USDA110 was able to grow to an OD_600_ &gt; 1.0 in 20 days in YEM (Fig. 1b), and its PHB content gradually accumulated up to 140 μg/OD_600_ in 12 days (Fig. 1c). In contrast, ΔphaR grew in YEM with less cell yield with an OD_600_ ≤ 0.5 in 12 days (Fig. 1b), and its PHB accumulation was observed at the highest level of 35 μg/OD_600_ after 4 days, but decreased later to leave only residual amounts after 12 days (Fig. 1c). USDA110 produces EPS when grown in YEM, and its EPS production was observed up to 12 μg/OD_600_ in 4 days, and decreased to about 5 μg/OD_600_ after 12 days. The EPS production of ΔphaR was nearly constant, around 10 μg/OD_600_ during the 12 day growth (Fig. 1d). Both USDA110 and ΔphaR produced similar intracellular glycogen, at 11.1 ± 1.14 and 11.7 ± 2.32 μg/OD_600_ in 12 days, respectively (data not shown).

The defects in *phaR* led to changes in stress tolerance. After incubation at 50 °C, ΔphaR demonstrated better survival than USDA110 on both TY and YEM plates (Additional file 2: Figure S1). In contrast, under the osmotic stress conditions with elevated concentrations of NaCl, sucrose, or glucose, ΔphaR grew similarly to USDA110 (data not shown). Surprisingly, when cultured on TY agar plates under microaerobic conditions, ΔphaR grew better than USDA110, whereas the two strains exhibited no difference in growth under aerobic conditions (Additional file 3: Figure S2).

Taken together, inactivation of *phaR* caused various changes in the phenotype of *B. diazoefficiens*, including reduced growth and decreased PHB accumulation, along with elevated production of EPS when grown in YEM. In addition, it enhanced tolerance to heat-shock and microaerobic stresses. These results imply that *phaR* may be involved in regulation of not only PHB accumulation but also pleiotropic cellular functions.

### Transcriptomic changes caused by inactivation of phaR

*B. diazoefficiens* USDA110 does not accumulate PHB in TY, and thus we can assume that in these growth conditions PhaR exerts its regulatory function to repress *phaP* genes, as PHB granules are not developing [12]. To test this possibility, USDA110 and ΔphaR were grown in TY, and total RNA was extracted and subjected to analyze transcriptomic changes.

**Fig. 2:**
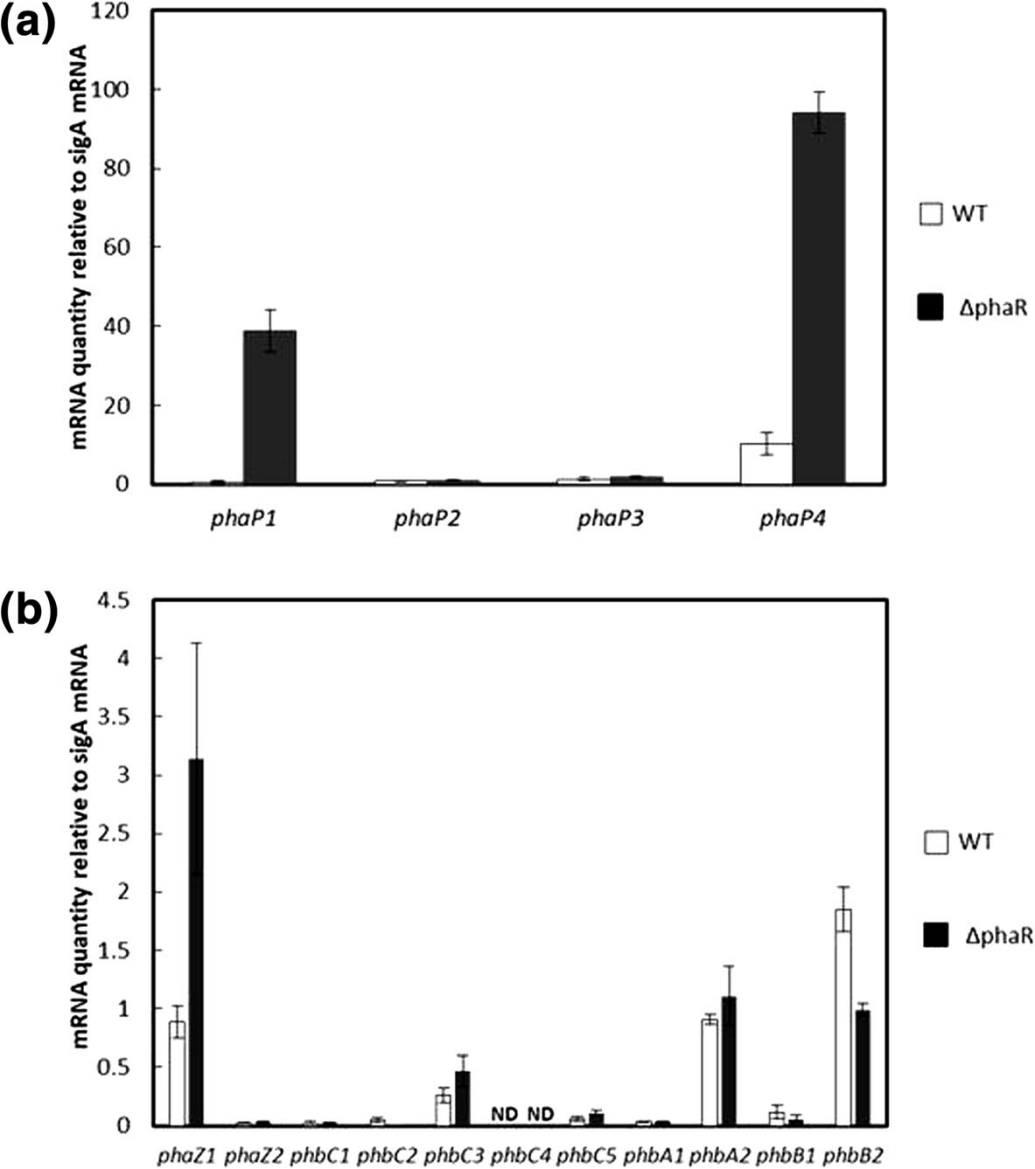
Transcription levels of *phaP* paralogs (**a**) and *phbA*, *phbB*, *phbC*, and *phaZ* paralogs (**b**) in USDA110 (WT, open bar) and ΔphaR (solid bar) grown in TY. Quantitative RT-PCR was performed as described in the main text to determine the transcription levels of the respective genes indicated along the horizontal axis, and data were normalized to the constitutive expression of *sigA* as the housekeeping sigma factor. Values are means ± standard deviation of three biologically independent experiments. ND: Not detected

Quantitative RT-PCR analysis was performed as previously reported [12]. First, we analyzed the expression of the four *phaP* paralogs, including *phaP1* (open reading frame bl15155), *phaP2* (bl1555), *phaP3* (bl16129), and *phaP4* (bl7395). As shown in (Fig. 2a), transcription of *phaP1* and *phaP4* increased in ΔphaR compared with that in USDA110. In contrast, transcription of both *phaP2* and *phaP3* remained low and did not change. Next, we analyzed the expression of the other eight genes involved in PHB synthesis, *phbA1* (blr3724), *phbA2* (bll0226), *phbB1* (bll3725), *phbB2* (bll0225), *phbC1* (blr2885), *phbC2* (blr3732), *phbC3* (bll4360), *phbC4* (bll4548), and *phbC5* (bll6073), as well as the two genes involved in PHB degradation, *phaZ1* (blr0908) and *phaZ2* (blr6703) (Fig. 2b). Expression of *phbA1*, *phbB1*, *phbC1*, *phbC2*, *phbC3*, and *phbC4* remained at relatively low levels, whereas expression of *phbA2* remained constitutive without obvious change. Interestingly, ΔphaR demonstrated an approximately twofold decrease in *phbB2* expression compared with that in USDA110, whereas expression of both *phbC5* and *phbC3* was elevated. With respect to the PHB-degrading genes, transcription of *phaZ1* was elevated in ΔphaR almost threefold over that of USDA110, whereas expression of *phaZ2* was only negligible. The quantitative RT-PCR experiments revealed that PhaR could be involved in regulation of not only *phaP1* and *phaP4* but also *phbB2* and *phaZ1* involved in PHB synthesis and degradation, respectively. In addition, inactivation of *phaR* led to various changes in phenotype, as described above. These results demonstrate that PhaR may regulate a large number of targets.

In order to assess the possibility, a transcriptome analysis as a screening test was performed by means of RNA sequencing on the RNA samples prepared from the cells grown in TY (Additional file 4: Table S2). As shown (Additional file 5: Figure S3), the distribution of RPKM values indicated that some genes exhibited altered expression with *phaR* inactivation. The genes in ΔphaR found to be induced in quantitative RT-PCR (Fig. 2) were also induced in RNA sequencing (Additional file 5: Figure S3). Nevertheless, RNA sequencing was more sensitive than quantitative RT-PCR, and it suggested that expression of 77 genes was elevated for more than fourfold in ΔphaR (Additional file 4: Table S2). Expression of *phaP1*, *phaP4*, and *phaZ1* was greatly increased in ΔphaR as compared with USDA110. We found that the transcription of *phaP5* (blr2887), which may encode an additional PhaP, prominently increased in ΔphaR. In addition, there was an elevation in the expression of the *cyoABCD* operon, which encodes the cytochrome *o* ubiquinol oxidase complex, one of the terminal oxidase complexes of the electron transport chain, which is usually induced in adaptation to microaerobic conditions [27]. Expression of the operon blr2367-blr2371 containing *exoZ*, encoding an acetyltransferase that transfers an acetyl group to EPS, was also increased, implying that this operon may be involved in the retained synthesis of EPS in ΔphaR (Fig. 1d). In ΔphaR, there were increases in the expression of *fixNOQP* (cbb3-type cytochrome *c* oxidase), *fixGHIS* (Fe-S-binding protein), *hemN2* (coproporphyrinogen III oxidase), *ppsA* (phosphoenolpyruvate synthase), and *nnrR* (regulator for nitric oxide metabolism), which are regulated by FixK_2_ [28]. In addition, the *napEDABC* operon (periplasmic nitrate reductase system) and *nirK* (copper-containing nitrite reductase) were also induced, which are reported to be induced by NnrR during adaptation to anaerobic conditions [29, 30]. These results suggest that inactivation of *phaR* may activate the FixK_2_ and NnrR regulons. Furthermore, some stress response genes also experienced increased transcription, including blr2761 and bll6069, whose products contain the Usp (universal stress protein) motif [31].

Unexpectedly, a large number of genes were suggested to decrease transcription in ΔphaR compared with USDA110 (Additional file 4: Table S2), as expression of 119 genes was lowered for more than fourfold in ΔphaR, including a putative operon for ABC transporter (blr0308-blr0312), a large gene cluster for polyketide synthesis including another ABC transporter (bll3369-bll3384), and the other genes related to flagellar formation and movement, such as *fliF1*, *fliR2*, *flhA2*, *flhB2*, *flgB2*, *flgC2*, *flgE2*, *flgG2*, *flgL2*, *motB2*, and *motC*. The results further suggest that PhaR may upregulate some active transport systems as well as cellular motility.

### DNA binding of PhaR

**Fig. 3:**
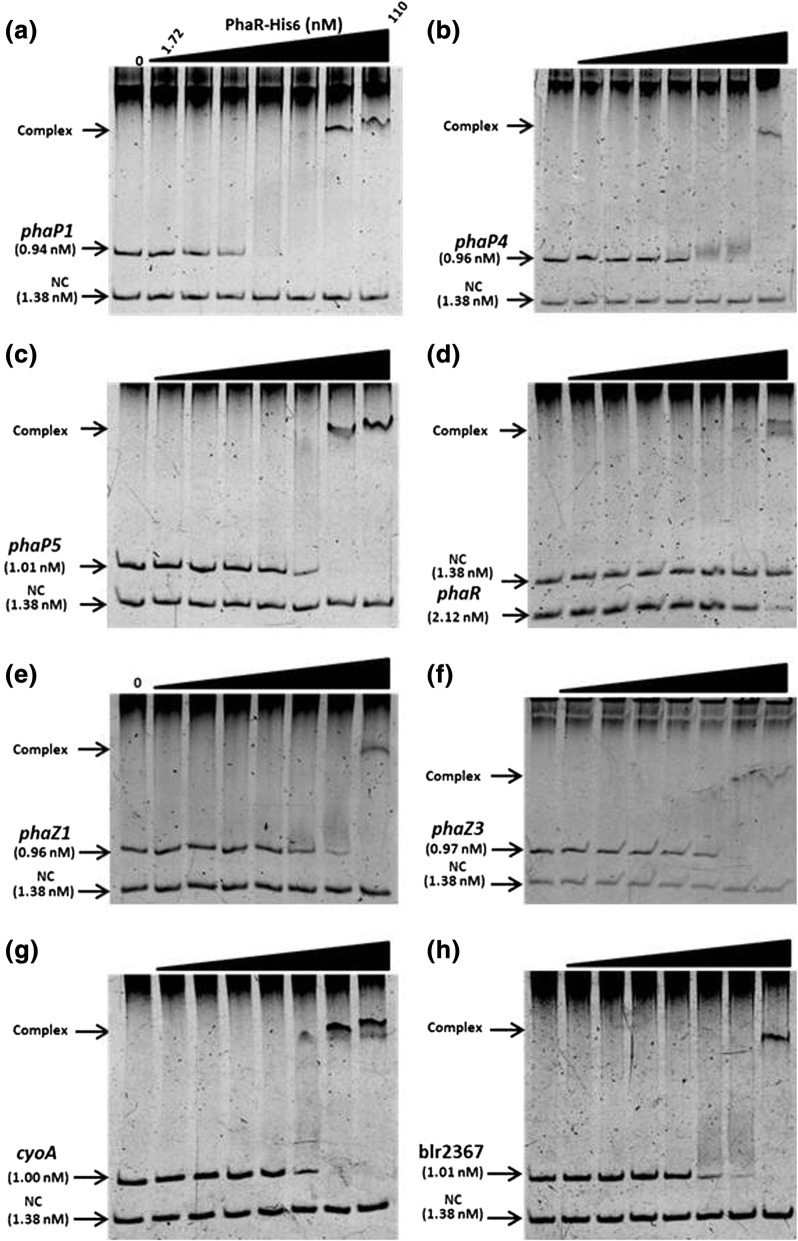
PhaR binding to DNA fragments containing the promoter regions of *phaP* paralogs (**a**–**c**), *phaR* (**d**), *phaZ* paralogs (e and f), and others (g and h). Various amounts of purified PhaR-His_6_ were incubated with a fixed amount of DNA fragments containing the promoter regions as indicated; (**a**) *phaP1* (bll5155), (**b**) *phaP4* (bll7395), (**c**) *phaP5* (blr2887), and (**d**) *phaR* (blr0227), (**e**) *phaZ1* (blr0908), (**f**) *phaZ3* (blr0899), (**g**) *cyoA* (blr0149), and (**h**) *blr2367*. The reactions were subjected to gel mobility shift assays as described in the main text. Each of the gels has eight lanes containing serially increased amounts of PhaR-His_6_ from left to right to give 0, 1.72, 3.44, 6.88, 13.8, 27.5, 55, and 110 nM, respectively. Positions for DNA-protein complex and free DNA fragments are indicated with arrowheads on the left side of the panels. As a negative control (NC), a constant amount of the DNA fragment corresponding to part of the *phaP1* coding region is included, which is another PCR fragment amplified using the primer pairs EMSA-phaP1-ORF-F/EMSA-phaP1-ORF-R (Additional file 1: Table S1)

Gel mobility shift assays using serially concentrated PhaR-His_6_ produced and purified in *E. coli* (Additional file 6: Figure S4) revealed that PhaR-His_6_ bound to the promoter region of *phaP1*, *phaP4*, *phaP5*, and *phaR* itself (Fig. 3a–d). Of the four DNA fragments, PhaR-His_6_ bound to the *phaP1* promoter most efficiently, as a decrease in the DNA fragment band was observed at 6.89 nM of PhaR-His_6_, and a distinct DNA-protein complex band appeared at 55 nM (Fig. 3a). However, for the *phaP4* promoter fragment, shifting of the band occurred at 13.8 nM and the complex appeared at 110 nM (Fig. 3b). For the fragment containing the *phaP5* promoter, which was suggested as an additional PhaR target after the transcriptome analysis described above, shifting of the band occurred at 27.8 nM and the complex was formed at 110 nM (Fig. 3c). These results indicated that PhaR-His_6_ bound to the promoter regions of *phaP1*, *phaP4*, and *phaP5*. In addition, PhaR-His_6_ was shown to bind to the DNA fragment of the *phaR* promoter (Fig. 3d), but only half of the DNA formed the complex at the highest PhaR-His_6_ concentration of 110 nM, suggesting a weaker affinity for the *phaR* promoter region. Next, gel mobility shift assays were performed as stated above with DNA fragments from the *phaZ1* and *phaZ3* (blr0899) promoters for PHB degrading enzymes (Fig. 3e and f, respectively). The *phaZ1* fragment shifted at 27.8 nM and completed the complex formation at 110 nM (Fig. 3e), whereas the *phaZ3* fragment shifted at 27.8 nM and completed the complex at 55 nM (Fig. 3f). These results suggested that these genes for PHB degrading enzymes could be regulated directly by PhaR.

Further, we assessed PhaR-His_6_ binding to the promoter regions of the possible PhaR targets suggested by the RNA sequencing transcriptome analysis (Fig. 3g and h). The DNA fragment from the promoter region of *cyoA* (*cyoABCD* operon) shifted at 27.8 nM of PhaR-His_6_ and completed the complex formation at 55 nM, suggesting higher affinity (Fig. 3g). The fragment from the promoter region of blr2367 for the *exoZ*-containing operon, shifted at 27.8 nM and completed the complex at 110 nM (Fig. 3h). In contrast, despite that its transcription was elevated more than fourfold in ΔphaR, no PhaR-His_6_ binding to the fragments containing promoter regions of *fixN*, *ppsA*, *nnrR*, and *hemN2* was detected, suggesting that PhaR may not directly regulate the expression of these genes (data not shown).

We performed additional gel mobility shift assays on the promoter regions of *phaP1* and *phaP4* in the presence of PHB (Additional file 7: Figure S5). In both cases, the PhaR-DNA complex formation decreased as the concentration of PHB increased. When 50 ng of PHB was added to the reaction mixture, binding of PhaR-His_6_ to the DNA was almost abolished, as the DNA bands appeared at the same position as the control without PHB, suggesting that PHB could inactivate DNA binding of PhaR-His_6_.

**Fig. 4:**
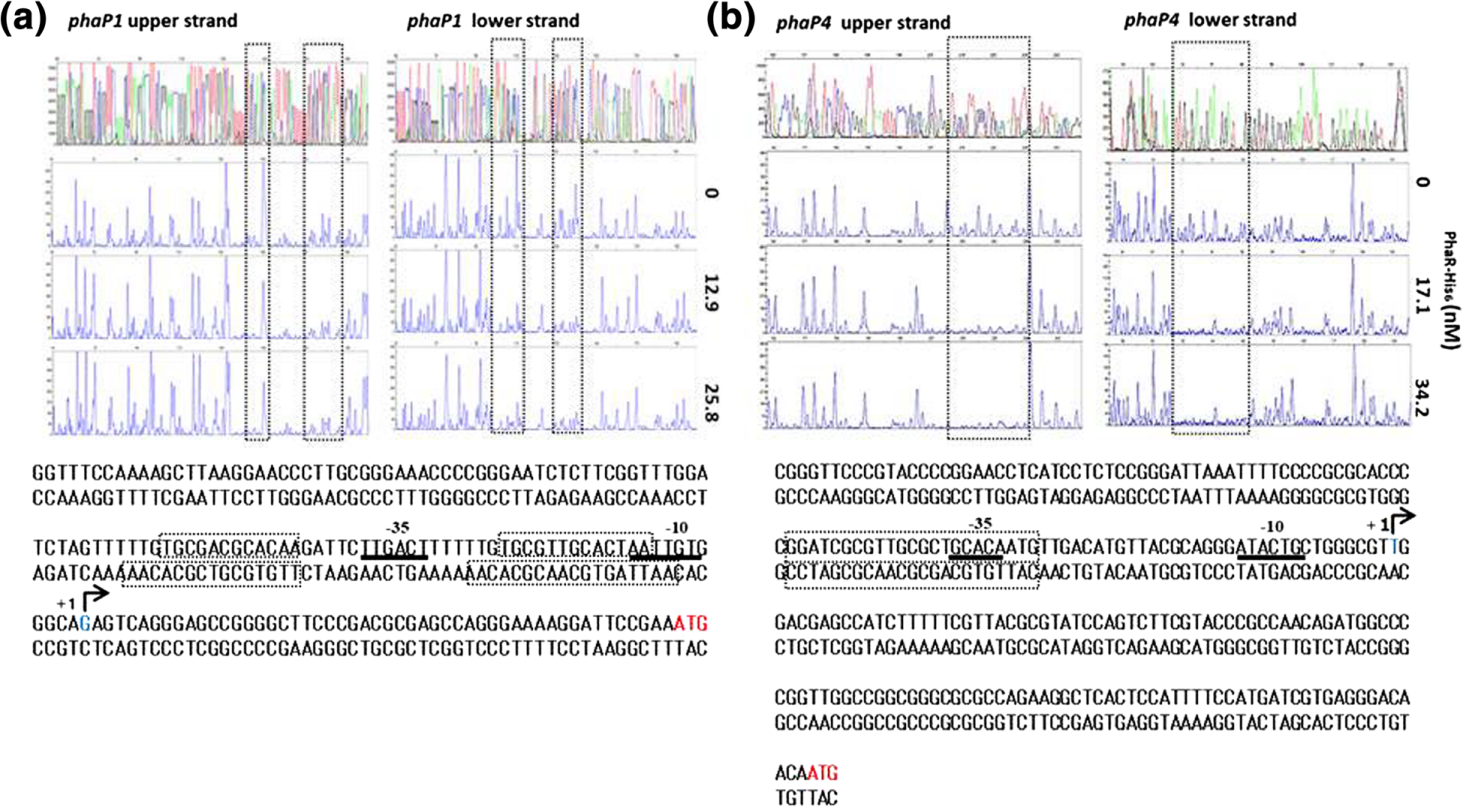
DNase I footprints of PhaR on the *phaP1* (**a**) and *phaP4* (**b**) promoter regions. DNaseI footprints of PhaR-His_6_ binding found in DNA fragment patterns are shown for the *phaP1* (**a**) and *phaP4* (**b**) promoter regions; the fragment patterns on upper and lower strands are in the left and right side of each panel, respectively. Each of the panels contain four fragment charts for respective upper and lower strands; from the top to down, the first is the sequencing ladders in four colors, the second is the negative control without PhaR-His_6_, and the third and fourth are the two different concentrations of PhaR-His_6_ as indicated. At the bottom of each panel, nucleotide sequences of the promoter regions of *phaP1* (**a**) and *phaP4* (**b**) are shown. The sequence stretches protected from DNase I digestion by PhaR-His_6_ binding are shown in hatched squares. Hocked arrowheads and the labels “+ 1” indicate the transcriptional start point (shown in blue letters in the upper strands). The −35 and − 10 regions are underlined and the ATG initiating codons are shown in red letters in the upper strands

In order to determine the PhaR-His_6_ binding sites, we performed DNase I footprint analyses on the *phaP1* and *phaP4* promoter regions (Fig. 4a and b, respectively). We found two sites occupied by PhaR-His_6_ within the *phaP1* promoter region and one in the *phaP4* promoter region. In addition, the respective transcription start points (+ 1) for the two promoters were determined by the 5′-RACE method using the RNA samples prepared from the ΔphaR cells grown in TY, and the corresponding − 10 and − 35 regions were deduced (Fig. 4). The two PhaR- binding sites with 12-mer sequences TGCGACGCACAA and TGCGTCGCACAA in the *phaP1* promoter region are located 6 bp upstream and downstream of the − 35 region, respectively, and the binding site with a sequence TGCGCTGCACAA in the *phaP4* promoter region overlaps the − 35 region. The presence of two binding sites within the *phaP1* promoter region may be responsible for its higher affinity to PhaR-His_6_ in vitro.

### Additional genes regulated by PhaR

**Fig. 5:**
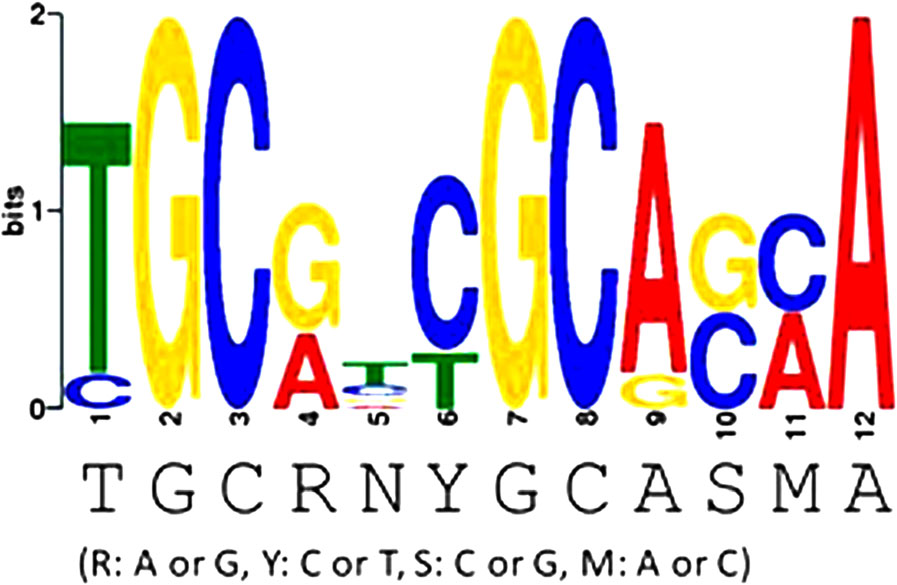
Prediction of putative PhaR-binding consensus sequence. Nucleotide sequences of the promoter regions of *phaP5*, *phaR*, *phaZ1*, *phaZ3*, the *cyoABCD* operon, and the *exoZ*-containing operon starting with blr2367 were aligned with three 12-mer sequences for PhaR-binding found in the *phaP1* and *phaP4* promoter regions by GLAM2 in MEME suit under the default settings for analysis to deduce a motif for PhaR binding. The graphical summary of GLAM2 analysis is shown with the consensus sequence TGCRNYGCASMA (R: A or G, Y: C or T, S: C or G, M: A or C) at the bottom

Since PhaR-His_6_ bound to the DNA fragments containing the additional six promoter regions, including those of *phaP5*, *phaR*, *phaZ1*, *phaZ3*, the *cyoABCD* operon, and the *exoZ*-containing operon starting with blr2367 (Fig. 3), we speculated that these promoter regions could share a sequence similarity. Nucleotide sequences of the six promoter regions were aligned with three 12-mer sequences of the PhaR-binding sites found in the *phaP1* and *phaP4* promoter regions (Fig. 4) by GLAM2 in MEME suit to deduce a motif of consensus sequence for PhaR binding as TGCRNYGCASMA (R: A or G, Y: C or T, S: C or G, M: A or C) (Fig. 5).

**Fig. 6:**
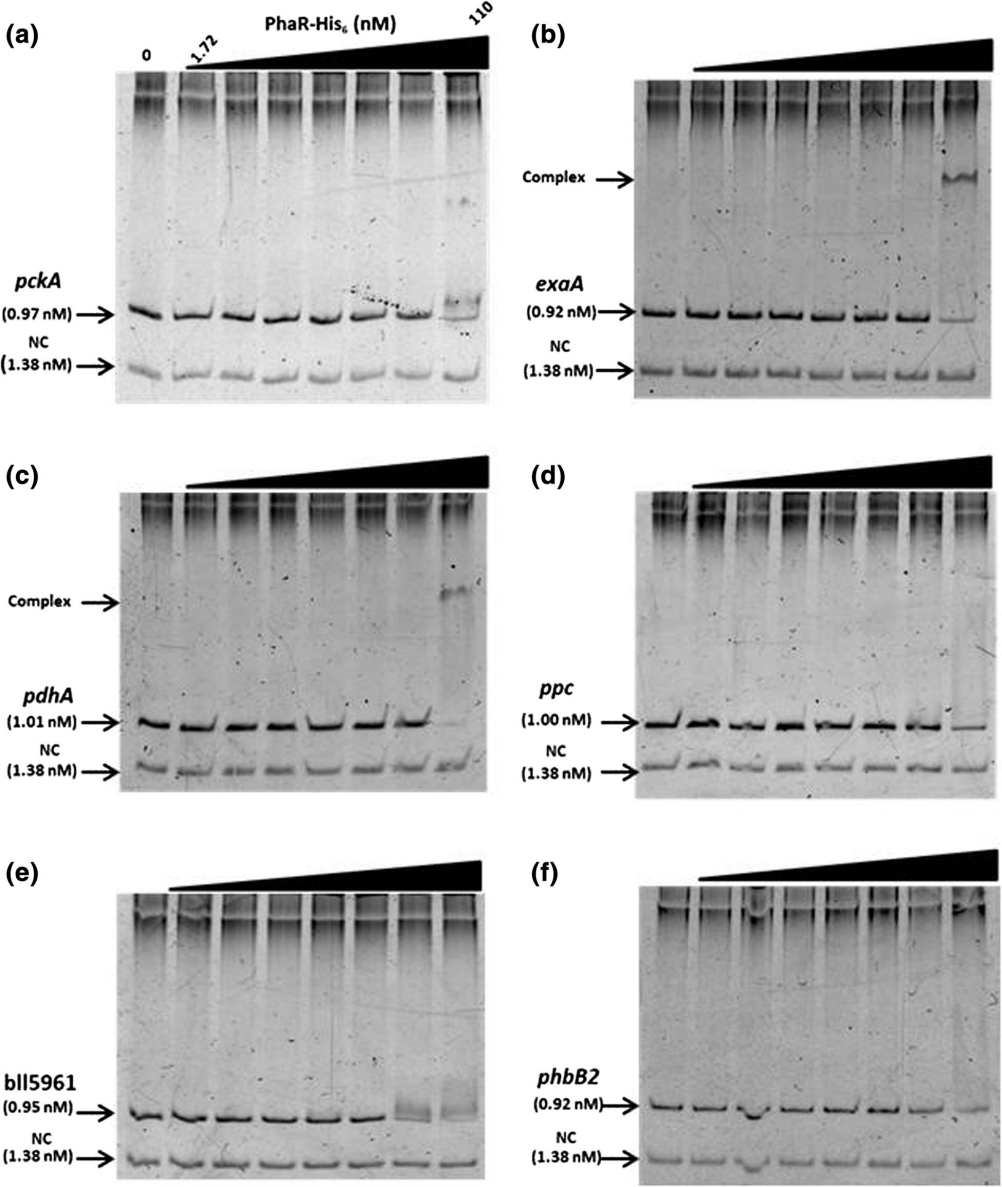
PhaR binding to DNA fragments of the promoter regions of *pckA* (bll8141) (**a**), *exaA* (blr6207) (**b**), *pdhAB* (bll4783-bll4779) operon (**c**), *ppc* (blr2955) (**d**), bll5961-blr5962 operon (**e**), and *phbB* (bll0225) (**f**). Experimental conditions and lane assignments are the same as shown in (Fig. 3)

We searched for sequences similar to the consensus sequence within the putative promoter regions of the genes whose transcription increased or decreased more than twofold in ΔphaR compared with USDA110 (Additional file 4: Table S2). Their putative promoter regions were selected on the basis of the possible transcription start points reported in recent studies [32]. We extracted 28 and 42 genes associated with the PhaR-binding consensus-like sequence, whose transcription increased (Table 2) and decreased (Table 3) in ΔphaR, respectively. Among the genes whose transcription was increased in ΔphaR (i.e., PhaR-repressed), the *ppc* (encoding phosphoenolpyruvate carboxylase) promoter region demonstrated a weaker affinity for PhaR-His_6_, as approximately half of the DNA band shifted only at the highest PhaR-His_6_ concentration of 110 nM (Fig. 6). However, among the genes whose transcription decreased (PhaR-activated), DNA fragments containing the promoter regions of bll5961 (encoding a putative regulatory protein of Crp family), *exaA* (quinoprotein ethanol dehydrogenase), *pckA* (phosphoenolpyruvate carboxykinase), *pdhA* (pyruvate dehydrogenase subunit alpha), and *phbB2* demonstrated some mobility shift at 110 nM of PhaR-His_6_ (Fig. 6). The results suggested that these consensus-sequence sites might have lower affinities to PhaR but possibly be involved in the regulation by PhaR.

**Table 2:** PhaR-repressed genes associated with a PhaR-binding consensus sequence

Gene or orf	PhaR binding site	USDA110 RPKM	ΔphaR RPKM	Fold change^a^	Description
*cyoA* (blr0149)	TGCGGCGCAGCA	22.29	77.7	3.49	cytochrome O ubiquinol oxidase subunit II
*phaZ3* (blr0899)	TGCAGTGCAGCA	32.68	89.48	2.74	poly(3-hydroxyalkanoate) depolymerase
*phaZ1* (blr0908)	CGCATCGCAGCA	129.32	542.25	4.19	poly(3-hydroxybutyrate) depolymerase
*rhlE* (bll1447)	TGCAGTGCAGAA	152.94	551.69	3.61	dead-box ATP-dependent RNA helicas
blr2242	TGCGCCGCAGCA	66.21	190.65	2.88	unknown protein
bll2363	TGCGCCGCACAA	374.44	1256.32	3.36	unknown protein
blr2367	TGCACCGCAGCA	55.14	347.34	6.30	acetyltransferase
bll2471	TGGATCGCACCA	86.78	512.16	5.90	hypothetical protein (Hemerythrin HHE cation binding region)
bll2537	CGCGTCGCAGCA	39.54	83.05	2.10	hypothetical protein
*phaP5* (blr2887)	TGCATCGCACAA	122.86	15,882.63	129.27	phasin
*ppc* (blr2955)	TGCGCTGCGGCA	46.27	93.71	2.03	PEP carboxylase
bll3794	TGCATTGCAGCG	104.59	404.72	3.87	hypothetical protein
blr4162	TGCATCGCACCA	79.46	931	11.72	hypothetical protein (cellulose synthase catalytic subunit protein)
bsr4236	TGCATTGCAACA	38.73	435.02	11.23	unknown protein
*nifR* (blr4486)	TGCAGTGCAGCA	112.16	225.25	2.01	nitrogen regulation protein
*metN* (blr4501)	TGCACCGCAACA	14.42	29.08	2.02	probable ABC transporter ATP-binding protein
bsr4726	TGCGGTGCACAC	157.38	1402.14	8.91	hypothetical protein
bll4785	CGCGCCGCACAA	90.88	279.72	3.08	transcriptional regulatory protein Fis family
*phaP1* (bll5155)	TGCAACGCACAA	215.67	10,738.12	49.79	phasin
bsr5273	TGCGGTGCATCA	45.13	222.49	4.93	hypothetical protein
bll5524	AGCAGTGCAGCA	106.54	220.06	2.07	hypothetical protein
blr5525	AGCAGTGCAGCA	23.4	46.85	2.00	hypothetical protein
blr5594	TGCGGCGCACAA	26.16	71.75	2.74	MFS permease
bll6290	TGCGGCGGACCA	146.99	458.11	3.12	two-component response regulator
blr6718	TGCCATGCAGCA	42.45	111.4	2.62	hypothetical protein
*nirK* (blr7089)	TGCGCTGCAACA	13.49	41.36	3.07	respiratory nitrite reductase
*phaP4* (bll7395)	TGCGCTGCACAA	489.78	2956.78	6.04	phasin
blr7872	TGCGCTGCAACA	6.30	20.63	3.27	HlyD family secretion protein

^a^Fold change: ΔphaR RPKM/USDA110 RPKM

**Table 3:** PhaR-activated genes associated with a PhaR-binding consensus sequence

Genes or orf	PhaR binding site	USDA110 RPKM	ΔphaR RPKM	Fold change^a^	Description
*phbB2* (bll0225)	TGCGCTGCACAC	154.34	74.52	2.07	acetoacetyl CoA reductase
*ragA* (bll0304)	TGCGACGCCGCA	8.95	2.29	3.91	two-component response regulator
blr0305	TGCGACGCCGCA	45.61	16.48	2.77	unknown protein
bll0805	CGCAACGCACAA	185.17	76.29	2.43	hypothetical protein
blr0806	CGCAACGCACAA	52.09	23.58	2.21	hypothetical protein
bll1416	TGCGCGGCAGCA	52.49	20.56	2.55	unknown protein
blr2204	TGCAGTCCAGAA	19.07	9.05	2.11	transcriptional regulatory protein AraC family
bll2446	TTCGCCGCAGAA	275.72	129.25	2.13	hypothetical protein
bsr2601	TGCACCGCAGCC	32.44	15.65	2.07	unknown protein
blr2810	TGCATTGCGCAA	15.88	7.35	2.16	aldo/keto reductase
bll2914	TGCGCTGGAGAA	25.07	9.61	2.61	probable amidase
bll3387	TGCGCCGCAACA	278.27	54.25	5.13	unknown protein
blr3795	TGCATTGCAGCG	40.09	19.03	2.11	ABC transporter HlyB/MsbA family
blr3904	TGCAGTGCTGCA	312.33	109.92	2.84	probable iron transport protein
blr4188	CGCAGTGCAGCA	28.85	12.98	2.22	hypothetical protein
bll4430	TGCAGCGCAGCA	104.34	38.21	2.73	hypothetical protein
bsr4431	TGCAGCGCAGCA	216.36	77.85	2.78	hypothetical protein
*pdhA* (bll4783)	TGCAGTGCGGCA	249.67	111.10	2.25	pyruvate dehydrogenase alpha subunit
bll4833	TGCGGCGCACCA	530.73	140.09	3.79	unknown protein
bsr4834	TGCGGCGCACCA	168.95	52.96	3.19	unknown protein
blr4841	TGCCGCGCACAA	30.64	14.59	2.10	unknown protein
bll4952	TGCATCGCACAA	27.07	7.50	3.61	NfeD protein homolog
bll5160	TGCGGCGCACAA	106.54	46.40	2.30	conserved hypothetical protein; putative alpha/beta-Hydrolases
bsl5321	CGCGGCGCAGCA	415.34	205.51	2.02	unknown protein
bll5335	TGCGCCGGACAA	35.29	16.85	2.09	putative thiolase
blr5540	TGCGGTGCCCAA	231.26	58.07	3.98	hypothetical protein
bsl5717	TGCGGCGCCCAA	109.78	34.94	3.14	hypothetical protein
bll5961	TGCGGTGCAACA	12.66	4.23	2.99	transcriptional regulatory protein Crp family
blr5962	TGCGGTGCAACA	40.98	7.40	5.54	ABC transporter ATP-binding protein
bll6121	TGCAGCGCACAA	21.88	9.39	2.33	probable sulfite oxidase
bll6206	CGCGCCGCACAA	74.19	35.28	2.10	hypothetical protein
*exaA* (blr6207)	CGCGCCGCACAA	93.32	23.85	3.91	probable quinoprotein ethanol dehydrogenase precursor
blr6443	TGCAATGCAACA	13.79	2.83	4.87	ABC transporter permease protein
blr6465	CGCGATGCACAA	39.03	14.29	2.73	putative steroid monooxygenase
bll6733	TGCGACGAAGCA	45.09	21.88	2.06	putative amidase
*pqqA* (bsr6735)	TGCAGTGCAACA	156.84	40.03	3.92	putative pyrroloquinoline quinone synthesis protein A
blr6837	CGCAGTGCAGCA	34.10	7.30	4.67	hypothetical protein
blr6886	CGCATTGCACAA	57.55	25.47	2.26	transcriptional regulatory protein MarR family
bll7487	TGCGGAGCACAA	456.33	176.20	2.59	unknown protein
bll7511	TGCAACGCAGAT	167.13	62.44	2.68	unknown protein
bll7663	TGCACCGCAGCA	57.90	24.14	2.40	unknown protein
*pckA* (bll8141)	TGCGACGCACAA	386.03	107.43	3.59	phosphoenolpyruvate carboxykinase

^a^Fold change: USDA110 RPKM/ΔphaR RPKM

## Discussion

In the present study, phenotypic changes resulting from *phaR* inactivation were investigated. There were no observable differences between ΔphaR and USDA110 grown in TY, whereas ΔphaR demonstrated decreased cell yield and diminished PHB accumulation in YEM (Fig. 1). These results suggest that altered PHB metabolism compromises the balance in intracellular carbon source usage. In fact, expression of *pdhAB* for acetyl-CoA synthesis was reduced in ΔphaR and transcription of *ppc* and *ppsA* increased, whereas those of *pckA* and *exaA* decreased. Together, these changes may cause a distortion in carbon metabolism. However, amounts of EPS produced in ΔphaR were approximately double those produced in USDA110 (Fig. 1), indicating that part of the energy and resources for PHB accumulation may be used for EPS production in ΔphaR. In addition, transcription of the operon containing *exoZ* increased in ΔphaR. Reduced EPS production was reported in an *exoZ* mutant strain of *S. meliloti* [33]. Therefore, the opposite effect may occur upon elevated expression of *exoZ* in ΔphaR, resulting in an increase in EPS production. Recently, the similar phenotypic changes were reported for another mutant with *phaR* inactivation [9]. On the other hand, we found ΔphaR improved thermal stress tolerance in both YEM and TY regardless of PHB accumulation (Additional file 2: Figure S1). It is known that PhaP expressed in *E. coli* exerted a molecular chaperone-like effect [34, 35]; in ΔphaR, PhaP1, PhaP4, and PhaP5 are produced in excess, which may restore proteins denatured under heat stress conditions. In addition, transcription of both blr2761 and bll6069 was elevated in ΔphaR, each of which encodes a protein containing the Usp motif, similar to *E. coli uspA*, which is involved in stress tolerance [31].

Interestingly, ΔphaR grew better under microaerobic conditions (Additional file 3: Figure S2). In ΔphaR, some genes that function under hypoxic conditions were enhanced (Additional file 4: Table S2), such as *fixNOQP*, *fixGHIS*, *napEDABC*, and *nirK*, and were increased, and these genes could be involved in enhancing growth and nitrogen fixation under anaerobic conditions. Nevertheless, the ΔphaR nodulation in soybean was nearly normal, with no obvious alteration in symbiotic nitrogen fixation under laboratory conditions (data not shown). The *cyoABCDE* operon of *E. coli* is induced under higher oxygen conditions [36], whereas in *R. etli* the *cyoABCD* operon was induced under hypoxic conditions to support growth [27]. Therefore, if the *cyoABCD* operon functions similarly in *B. diazoefficiens* as it does in *R. etli*, the increased expression of the *cyoABCD* operon in ΔphaR may explain its better growth under the microaerobic conditions.

PhaR could directly repress transcription of various genes; at least 28 of the genes may be negatively regulated and associated with a PhaR-binding site, including the *cyoABCD* operon, the operon involved in EPS production that contains *exoZ*, and *ppc* (Table 2). Inactivation of *phaR* also led to downregulation of a large number of genes, suggesting PhaR may function as a positive regulator; at least 42 of the genes may be associated with a PhaR-binding site (Table 3). Some genes involved in carbon metabolism, such as the *pdhAB* operon, *pckA*, and *exaA* could be upregulated through PhaR binding to their promoter regions (Fig. 6). In addition, PhaR binds to the promoter region of *phbB2* (Fig. 6), previously shown to be important for PHB synthesis [12], the transcription of which decreased twofold in ΔphaR (Fig. 2). In *R. etli*, PhaB disappeared in the *aniA* (a *phaR* homolog) mutant [18], implying direct involvement of PhaR in the production of PhbB2. In any case, further studies are required to understand the mechanism of PhaR in transcriptional activation.

We found two PhaR-His_6_ binding sites in the *phaP1* promoter and one in the *phaP4* promoter, as determined by DNase I footprint analyses (Fig. 4). The gel mobility shift assays indicated a higher PhaR-His_6_ affinity for the *phaP1* promoter than for the *phaP4* promoter (Fig. 3), suggesting that PhaR could exert tighter control over *phaP1* than *phaP4*. In a previous report, transcriptional activity of the *phaR* promoter in a *phaR* mutant doubled over that of the wild-type strain of *B. diazoefficiens* [9]. In the present study, the gel mobility shift assays revealed that PhaR-His_6_ binds to the *phaR* promoter region inefficiently (Fig. 3), implying possible leakiness in its own repression, allowing a constitutive level of PhaR expression in the cell. We demonstrated that DNA binding of PhaR-His_6_ was abolished in the presence of PHB in vitro (Additional file 7: Figure S5). These results were in agreement with the previous finding in *Paracoccus denitrificans* [37] and our previous observation that PhaR competed for PHB binding with PhaPs and was released from PHB as concentrations of PhaPs increased [12]. The liberated PhaR then binds DNA again to repress the PhaP genes. In our previous studies, PhaP4 showed a higher affinity for PHB than PhaP1 [12]. It is suggested that PhaR could control *phaP1* more tightly than *phaP4* (Fig. 3). Therefore, given its higher affinity for PHB, PhaP4 may be readily produced to release PhaR from PHB. It is possible that PhaP5 may also be play an important role, as it was induced almost fivefold over *phaP4* in ΔphaR (Table 2). Since PhaP1, PhaP4, and PhaP5 were all overexpressed in ΔphaR, PHB could be covered immediately by these PhaPs, thereby inhibiting the expansion of PHB granules. In addition, increasing expression of *phaZ1* and *phaZ3* could result in degradation of PHB. Previous in vitro experiments revealed that PhaPs on the surface of PHB granules could contribute to activity of PhaZ enzymes, and the possible interaction between PhaPs and PhaZ enzymes may have an effect on the decrease of accumulated PHB [38, 39].

In *Rhodobacter sphaeroides*, PhaR repressed transcription of *phaZ* for PHB degradation [40]. In the present study, we found that transcription of *phaZ1* in ΔphaR increased about fourfold over that in USDA110 (Table 2) and PhaR-His_6_ bound to the *phaZ1* promoter region in vitro (Fig. 3). Furthermore, a third gene for PHB degradation, *phaZ3*, was found; its expression was elevated by inactivation of *phaR* (Table 2), and PhaR-His_6_ binding to its promoter region was demonstrated in vitro (Fig. 3). Therefore, PhaR of *B. diazoefficiens* could negatively and directly control transcription of the two PHB degrading genes, *phaZ1* and *phaZ3*. PhaZ3 has the esterase PHB depolymerase motif, whereas PhaZ1 and PhaZ2 have the PHB depolymerase C-terminus motif (data not shown). In addition, a blast search against the genome of *R. eutropha* strain H16 revealed that PhaZ3 shares a homology to PhaZ7 of H16 (E-value = 5e–16). It was demonstrated that induction of *phaZ7* in a recombinant *E. coli* strain greatly reduced PHB accumulation [41]. Therefore, PhaZ3 may play an important role in PHB degradation in *B. diazoefficiens*.

The transcriptome analysis revealed an additional PhaP gene, *phaP5*, the expression of which was prominent in ΔphaR compared with USDA110 (Additional file 4: Tables 2). PhaP5 possesses the phasin 2 motif, similar to that found in the other four PhaPs. In the present study, we demonstrated PhaR-His_6_ binding to the promoter regions of these PhaP genes using gel mobility shift assays (Fig. 3). It is likely that PhaR binds to the promoter regions of *phaP1*, *phaP4*, and *phaP5* and represses their transcription under non-PHB-accumulating conditions. These results suggested that PhaP5 may play an important role in PHB accumulation together with PhaP1 and PhaP4.

It was also revealed that some of the genes regulated by FixK_2_, a global transcriptional activator for adaptation to microaerobic conditions, increased their expression in ΔphaR, PhaR-His_6_ may bind to the *fixK2* promoter region. However, a DNA fragment of the *fixK2* promoter region exhibited no interaction with PhaR-His_6_ under the conditions used in the present study (data not shown). When cultured under microaerobic conditions, transcription of *fixK2* increased in a mutant strain of *B. diazoefficiens* lacking *phaR* [9]. In the present study, however, transcription of *fixK2* in ΔphaR was not elevated significantly (Additional file 4: Table S2), and no PhaR-binding site was predicted in the promoter region of *fixK2*. Nevertheless, FixK_2_ is activated in response to a moderate decrease in oxygen concentration [28]. As described above, we found that transcription of both the *napEDABC* operon and *nirK*, which function in the uptake and reduction of nitric acid under anaerobic conditions, was elevated in ΔphaR [29, 30]. This implies that *phaR* inactivation may make the intracellular environment less aerobic as the metabolic circuit is modulated to accelerate respiration to consume more oxygen, and thus available oxygen could decrease to activate FixK_2_ for induction of its targets. PhbC3 could be active as a homodimer catalyzing PHB polymerization, whereas PhbC5 may bind to PhbC3 to form an inactive heterodimer [9]. In the present study, we found that PhaR may not directly control *phbC3* and *phbC5*. It was previously suggested that FixK_2_ may be involved in the induction of *phbC5* [9]. Expression of *phbC5* in ΔphaR appeared to increase slightly (Fig. 2), which may be one of the additional reasons why PHB did not accumulate in ΔphaR (Fig. 1).

## Conclusions

PhaR of *B. diazoefficiens* USDA110 is a DNA-binding transcription factor that is originally known to control the PHB granule stabilization in response to the intracellular levels of PHB. It was found that inactivation of *phaR* in USDA110 led to pleiotropic changes in cellular processes not only in PHB accumulation. It was demonstrated that PhaR regulated transcription of its various target genes, binding to their promoter regions that contain the relaxed consensus sequence, TGCRNYGCASMA (R: A or G, Y: C or T, S: C or G, M: A or C). These results suggest that PhaR could regulate a large number of genes to coordinate metabolism holistically in response to PHB accumulation.

## Additional files

Additional file 1: **Table S1.** Oligonucleotides used in this study. Names of oligonucleotides and their nucleotide sequences are listed. (PDF 66 kb)

Additional file 2: **Figure S1.** Heat stress resistance of *B. diazoefficiens*. *B. diazoefficiens* strains USDA110 (WT) and ΔphaR were pre-cultured in TY (a) and in YEM (b) for 2 and 12 days, respectively, and then diluted to yield OD600 = 0.1 in respective fresh media. For the heat shock stress experiments, the diluted cultures were incubated with shaking at 160 rpm at 50 °C for 5 and 10 min, respectively. After incubation, the cultures were serially diluted 1:10 for 5 times. Each of the diluted aliquots (5 μL) were spotted from left to right on to PSY plates and incubated at 28 °C for 1 week for colony formation. (PDF 169 kb)

Additional file 3: **Figure S2.** Colony formation of *B. diazoefficiens* under aerobic (a) and microaerobic (b) conditions. Cultures of *B. diazoefficiens* strains USDA110 (WT) and ΔphaR (OD600 = 0.1) were serially diluted 10 times, spotted on to TY plates, and grown under aerobic (a) and anaerobic (b) conditions at 28 °C. Pictures were taken after 5 (a) and 19 (b) days of incubation. (PDF 340 kb)

Additional file 4: **Table S2.** Summary of RNA sequencing analysis to compare transcriptome of USDA110 and ΔphaR. This is an excel spreadsheet summarizing the results of RNA sequencing analysis. (XLSX 1269 kb)

Additional file 5: **Figure S3.** Overview of RNA sequencing analysis data quality. (a) Comparison of USDA110 (WT) and ΔphaR RPKM values. (b) Comparison of log2 transformed data of quantitative RT-PCR and RNA sequencing. Fold change refers to the relative expression in ΔphaR to USDA110 (WT). (PDF 146 kb)

Additional file 6: **Figure S4.** Purification of PhaR-His_6_. PhaR-His_6_ was purified by Ni-Co affinity chromatography and its purity analyzed using a 12% SDS-polyacrylamide gel. Lane M, molecular weight markers; lane 1, flow through fraction; lane 2, wash fraction; lanes 3–5, eluted fractions with 40, 100, and 200 mM imidazole, respectively. The purified PhaR-His_6_ is found at approximately 22.6 kDa. (PDF 128 kb)

Additional file 7: **Figure S5.** PhaR binding to DNA fragments containing the promoter regions of phaP paralogs in the presence of PHB. A fixed amount of DNA fragments containing the phaP1 (a) and phaP4 (b) promoter regions were incubated with the indicated amount of PhaR-His_6_ and various amounts of PHB (0 to 50 ng per reaction as indicated). The control lane contained neither PhaR-His_6_ nor PHB. The negative control (NC) DNA is the same as Fig. 3. (PDF 63 kb)

## Abbreviations

EPS: Exopolysaccharides
Km: Kanamycin
LB: Lysogeny broth medium
PHA: Polyhydroxyalkanoate
PhaP: Phasin protein
PHB: Poly-3-hydroxybutyrate
Pm: Polymyxin B
PSY: Peptone salts yeast extract medium
YEM: Yeast extract mannitol medium
