# Supplementary material for: Bradyrhizobium diazoefficiens USDA110 PhaR functions for pleiotropic regulation of cellular processes besides PHB accumulation
Source: BMC Microbiol. 2018 Oct 24;18:156. doi: 10.1186/s12866-018-1317-2 (PMC6201568; doi:10.1186/s12866-018-1317-2)
Supplement: Supplementary file 1 — Table S1. Oligonucleotides used in this study. Names of oligonucleotides and their nucleotide sequences are listed. (PDF 66 kb) [file 12866_2018_1317_MOESM1_ESM.pdf]

Table S1. Oligonucleotides used in this study.

Oligonucleotide	Sequence (5´-3´) *
FFphaR	CCCAAGCTTCCGACCGGGGTGC
FRphaR	CGACATCCGCTCGAGGCGTTCGTTATAGAGCCGGCGGTTCGC
RFphaR	GCGAACCGCCGGCTCTATAACGAACGCCTCGAGCGGATGTCG
RRphaR	CTAGTCTAGACGCATTGGCGGACAGGTCCA
phaR-c-F	CTGACGATGACGACATCGTCTG
phaR-c-R	GAGATTGCGGTATCCGTCCTG
RTphbA2-F	AAGAAGGCCGGCTGGAA
RTphbA2-R	CCATTGACGTTGACCTTGGA
RTphbB2-F	CCGAAGGCGTGAAGAAGGT
RTphbB2-R	GAACAGCGAGCCGAGATTG
RTphbC3-F	ATGACCGCGTCGAACCA
RTphbC3-R	GGCACCTTGACCTTGGAGA
RTphbC5-F	CCGCAAAATTCCCTGGTC
RTphbC5-R	CATCCCTGTCCTTCGCA
RTphaZ1-F	CCGAAGCAACGCACACA
RTphaZ1-R	ATCCTCGGCACGATTTCC
RTphaZ2-F	GGCACATCAAGCAGCACA
RTphaZ2-R	AGATCCATCACCGCGAAA
RTphaP1-F	ACGGCGACTACACCAAGAAG
RTphaP1-R	GAAGGTCTCGTAGGCGGAAC
RTphaP2-F	TCGCTTTTACCGAGCAGAA
RTphaP2-R	GTGAACTGGCTACGCAGGA
RTphaP3-F	ATTACGGCACCAAGGTCATC
RTphaP3-R	GTGGAGAGGTTCACGAGGTC
RTphaP4-F	GTGCGACTGATCCATTCTCC
RTphaP4-R	GTCCTTGAACTTGGCGTAGC
RTsigA-F	CAGGCGAAGGACAAGGAAAA
RTsigA-R	CGTCGGACAGATCGAGCAA
phaP1S2	ATGGAAGCGCAGACCGACTTC
phaP1S1	GTGAAGTCGCTGGACAAG
phaP1A1	GAAAGCTTCTCGACGAAGGAC
phaP1A2	GTCTTCGAACGACTTCTTGG
RT-phaP1-phos	GAATGCCTGCTTGGC

phaP4S2	CATCTGGACTTCACCCAG
phaP4S1	CATGAAGGCCAATACGACC
phaP4A1	ATCAGCTTGGCGGTGTACTC
phaP4A2	ACGAGAACACGGCCTCAATG
RT-phaP4-phos	GAAGGTCTCGAGCTG
pET28b-inverseNcoI-F	GCCCATGGTATATCTCCTTC
pET28b-inverseBamHI-R	GGATCCGAATTCGAGCTCCG
pET28b-phaR-gib-F	AGATATACCATGGGCATGGCGAAATCAGACCAACC
pET28b-phaR-gib-His6-R	CTCGAATTCGGATCCCTCTTCCTTCTTCGACATCCGC
EMSA-phaP1-ORF-F	CTCCAGGCAATCGCCAG
EMSA-phaP1-ORF-R	TCTCGACCGGCTTGAATGC
EMSA-phaP1-F-2	CTAAGGTGATGTTCTCAAACGGC
EMSA-phaP1-R-2	GAACTGCTCTTTCCCGTAGCTCTG
EMSA-phaP4-F	TCCGGGGGATGTCATTC
EMSA-phaP4-R	GTACGAAGACTGGATACGC
EMSA-phaR-F	ACGTTTTTGCTGCCCGATTAAC
EMSA-phaR-R	CTCTCACTTGCAAGACGCT
EMSA-cyoA-F	AGTCGAAGAATTCCGAGGTGCG
EMSA-cyoA-R	GGGCCGAGCCTTTGAGAATG
EMSA-exoZ-operon-F	AATGCGAGCTGGCGTGTTGAC
EMSA-exoZ-operon-R	TTGAAAGACGCTCTACGATTC
EMSA-phaP5-F	ACGGCAAGACCCACGACATTGTC
EMSA-phaP5-R	GGGGTTCTCTCCATCCTCAATG
EMSA-phaZ1-F-2	AACGGCTCGCTGCTGTCCAG
EMSA-phaZ1-R-2	GGATTGAGGGGATTCTGAAACAGG
EMSA-pckA-F	GCCCAGGGAATTGTGCGTC
EMSA-pckA-R	CGAAGGCACCGTTGCGCAC
EMSA-phaZ3-F	GGCATTGCGGTTAATGCGTGC
EMSA-phaZ3-R	GCGCGGCATCTGCAACTGC
EMSA-blr5962-F	ATCCCGAAGCCAGCCGCAC
EMSA-blr5962-R	CCGGTGATGGCGCTTTCTTTGG
EMSA-phbB-F	CCAATGCAACACGTGCCATATCG
EMSA-phbB-R	CTTGCCACGCTGTGCATCG
EMSA-ppc-F	GGCGTGATCGGCAATATCATCTGG
EMSA-ppc-R	CTCGCTCCCCTGGTTCGAG
EMSA-pdhA-F	CACAAGATGATGCTCGATCACTACC

EMSA-pdhA-R	CCGGGTCAGAGAAAACTTTCGTCC
EMSA-exaA-F	CTCACCAAGAGCTCCGATTCG
EMSA-exaA-R	CATCCGTTCCTCCAAATATGCGC
EMSA- fixK2-F	CTACGTGATCGACTACAAGATGCC
EMSA- fixK2-R	CTCCGTTGTGATGACGCATTGGTAC
FAM-phaP1-F	CTAAGGTGATGTTCTCAAACGGC
FAM-phaP1-R	GAACTGCTCTTTCCCGTAGCTCTG
FAM-phaP4-F	TCCGGGGGATGTCATTC
FAM-phaP4-R	GTACGAAGACTGGATACGC

*Restriction sites are underlined.
