# Supplementary material for: Bradyrhizobium diazoefficiens USDA110 PhaR functions for pleiotropic regulation of cellular processes besides PHB accumulation
Source: BMC Microbiol. 2018 Oct 24;18:156. doi: 10.1186/s12866-018-1317-2 (PMC6201568; doi:10.1186/s12866-018-1317-2)
Supplement: Supplementary file 2 — Figure S1. Heat stress resistance of B. diazoefficiens. B. diazoefficiens strains USDA110 (WT) and ΔphaR were pre-cultured in TY (a) and in YEM (b) for 2 and 12 days, respectively, and then diluted to yield OD600 = 0.1 in respective fresh media. For the heat shock stress experiments, the diluted cultures were incubated with shaking at 160 rpm at 50 °C for 5 and 10 min, respectively. After incubation, the cultures were serially diluted 1:10 for 5 times. Each of the diluted aliquots (5 μL) were spotted from left to right on to PSY plates and incubated at 28 °C for 1 week for colony formation. (PDF 169 kb) [file 12866_2018_1317_MOESM2_ESM.pdf]

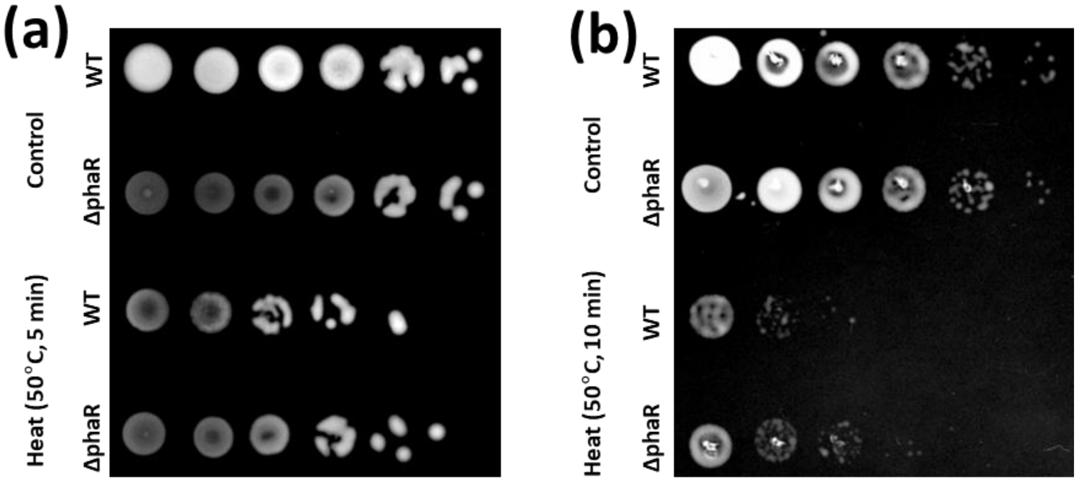

Fig. S1. Heat stress resistance of *B. diazoefficiens. B. diazoefficiens* strains USDA110 (WT) and ΔphaR were pre-cultured in TY (a) and in YEM (b) for 2 and 12 days, respectively, and then diluted to yield OD_{600} = 0.1 in respective fresh media. For the heat shock stress experiments, the diluted cultures were incubated with shaking at 160 rpm at 50 °C for 5 and 10 min, respectively. After incubation, the cultures were serially diluted 1:10 for 5 times. Each of the diluted aliquots (5 μL) were spotted from left to right on to PSY plates and incubated at 28 °C for 1 week for colony formation.
