# Supplementary material for: Bradyrhizobium diazoefficiens USDA110 PhaR functions for pleiotropic regulation of cellular processes besides PHB accumulation
Source: BMC Microbiol. 2018 Oct 24;18:156. doi: 10.1186/s12866-018-1317-2 (PMC6201568; doi:10.1186/s12866-018-1317-2)
Supplement: Supplementary file 3 — Figure S2. Colony formation of B. diazoefficiens under aerobic (a) and microaerobic (b) conditions. Cultures of B. diazoefficiens strains USDA110 (WT) and ΔphaR (OD600 = 0.1) were serially diluted 10 times, spotted on to TY plates, and grown under aerobic (a) and anaerobic (b) conditions at 28 °C. Pictures were taken after 5 (a) and 19 (b) days of incubation. (PDF 340 kb) [file 12866_2018_1317_MOESM3_ESM.pdf]

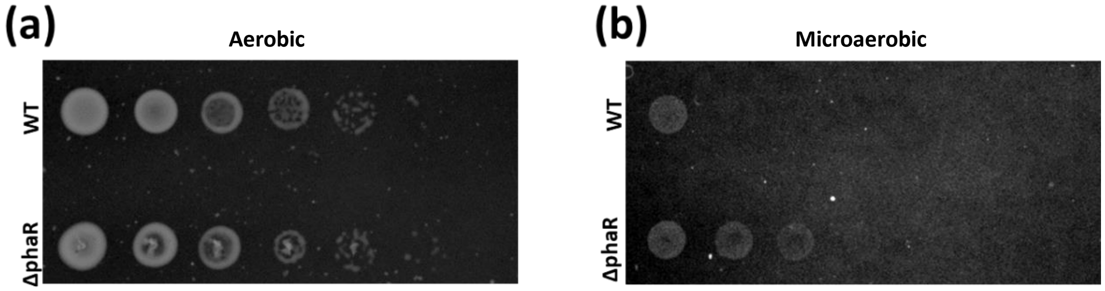

Fig. S2. Colony formation of *B. diazoefficiens* under aerobic (a) and microaerobic (b) conditions. Cultures of *B. diazoefficiens* strains USDA110 (WT) and ΔphaR (OD_{600} = 0.1) were serially diluted 10 times, spotted on to TY plates, and grown under aerobic (a) and anaerobic (b) conditions at 28 °C. Pictures were taken after 5 (a) and 19 (b) days of incubation.
