# Supplementary material for: Bradyrhizobium diazoefficiens USDA110 PhaR functions for pleiotropic regulation of cellular processes besides PHB accumulation
Source: BMC Microbiol. 2018 Oct 24;18:156. doi: 10.1186/s12866-018-1317-2 (PMC6201568; doi:10.1186/s12866-018-1317-2)
Supplement: Supplementary file 5 — Figure S3. Overview of RNA sequencing analysis data quality. (a) Comparison of USDA110 (WT) and ΔphaR RPKM values. (b) Comparison of log2 transformed data of quantitative RT-PCR and RNA sequencing. Fold change refers to the relative expression in ΔphaR to USDA110 (WT). (PDF 146 kb) [file 12866_2018_1317_MOESM5_ESM.pdf]

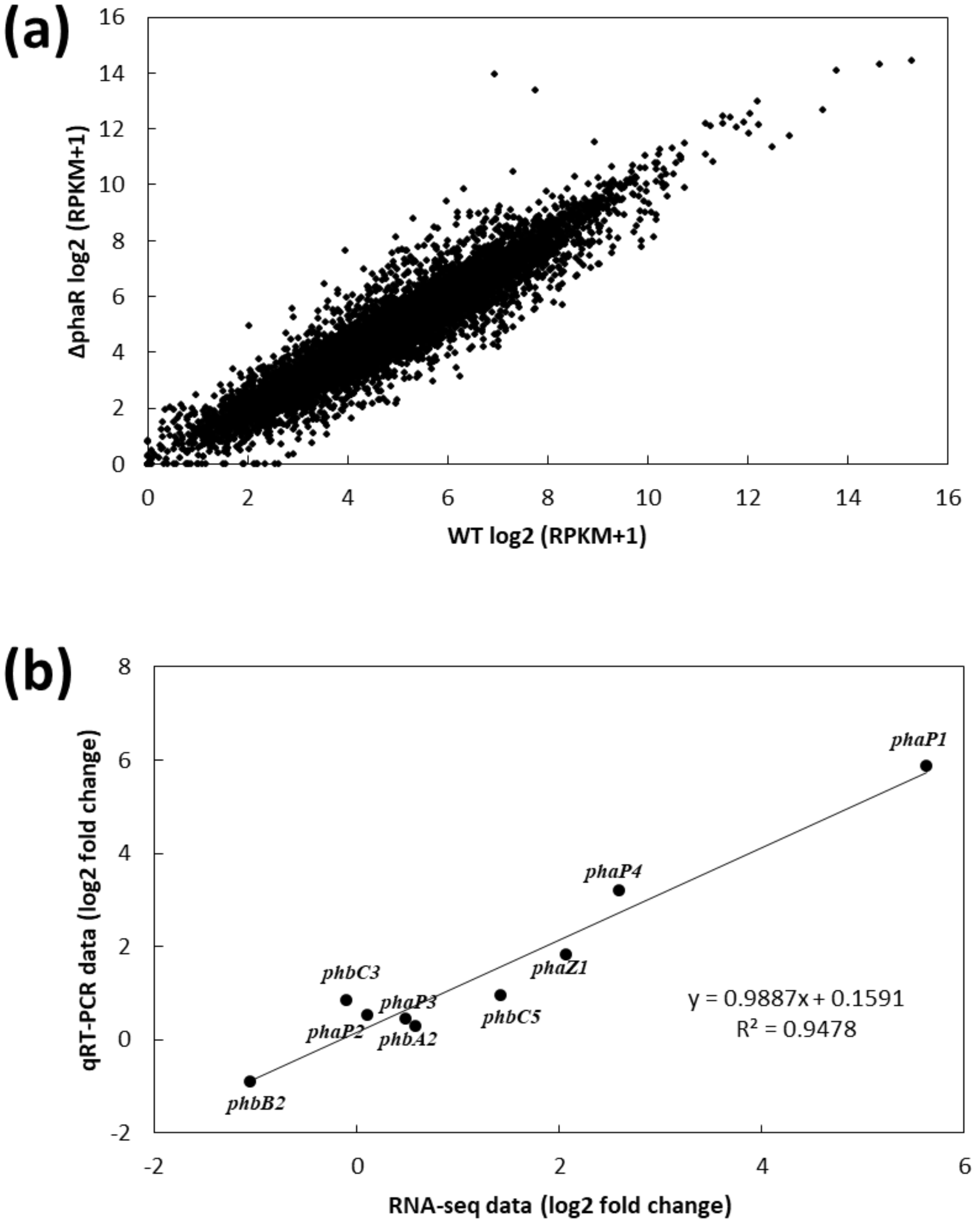

Fig. S3. Overview of RNA sequencing analysis data quality. (a) Comparison of USDA110 (WT) and ΔphaR RPKM values. (b) Comparison of log2 transformed data of quantitative RT-PCR and RNA sequencing. Fold change refers to the relative expression in ΔphaR to USDA110 (WT).
