# Supplementary material for: Bradyrhizobium diazoefficiens USDA110 PhaR functions for pleiotropic regulation of cellular processes besides PHB accumulation
Source: BMC Microbiol. 2018 Oct 24;18:156. doi: 10.1186/s12866-018-1317-2 (PMC6201568; doi:10.1186/s12866-018-1317-2)
Supplement: Supplementary file 6 — Figure S4. Purification of PhaR-His6. PhaR-His6 was purified by Ni-Co affinity chromatography and its purity analyzed using a 12% SDS-polyacrylamide gel. Lane M, molecular weight markers; lane 1, flow through fraction; lane 2, wash fraction; lanes 3–5, eluted fractions with 40, 100, and 200 mM imidazole, respectively. The purified PhaR-His6 is found at approximately 22.6 kDa. (PDF 128 kb) [file 12866_2018_1317_MOESM6_ESM.pdf]

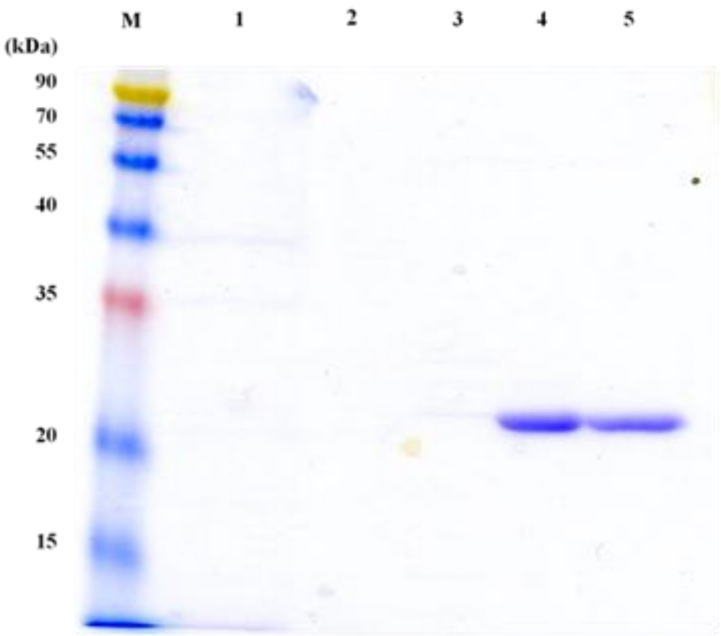

Fig. S4. Purification of PhaR-His_6. PhaR-His_6 was purified by Ni-Co affinity chromatography and its purity analyzed using a 12% SDS-polyacrylamide gel. Lane M, molecular weight markers; lane 1, flow through fraction; lane 2, wash fraction; lanes 3–5, eluted fractions with 40, 100, and 200 mM imidazole, respectively. The purified PhaR-His_6 is found at approximately 22.6 kDa.
