# Supplementary material for: Bradyrhizobium diazoefficiens USDA110 PhaR functions for pleiotropic regulation of cellular processes besides PHB accumulation
Source: BMC Microbiol. 2018 Oct 24;18:156. doi: 10.1186/s12866-018-1317-2 (PMC6201568; doi:10.1186/s12866-018-1317-2)
Supplement: Supplementary file 7 — Figure S5. PhaR binding to DNA fragments containing the promoter regions of phaP paralogs in the presence of PHB. A fixed amount of DNA fragments containing the phaP1 (a) and phaP4 (b) promoter regions were incubated with the indicated amount of PhaR-His6 and various amounts of PHB (0 to 50 ng per reaction as indicated). The control lane contained neither PhaR-His6 nor PHB. The negative control (NC) DNA is the same as Fig. 3. (PDF 63 kb) [file 12866_2018_1317_MOESM7_ESM.pdf]

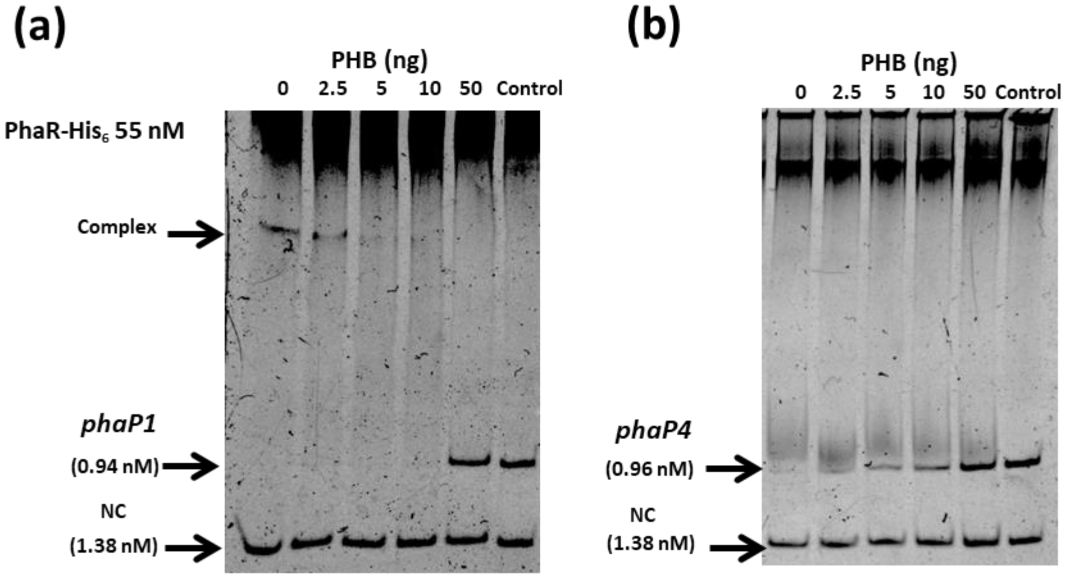

Fig. S5. PhaR binding to DNA fragments containing the promoter regions of *phaP* paralogs in the presence of PHB. A fixed amount of DNA fragments containing the *phaP1* (a) and *phaP4* (b) promoter regions were incubated with the indicated amount of PhaR-His$_6$ and various amounts of PHB (0 to 50 ng per reaction as indicated). The control lane contained neither PhaR-His$_6$ nor PHB. The negative control (NC) DNA is the same as Fig. 3.
